# Targeted CD7 CAR T-cells for treatment of T-Lymphocyte leukemia and lymphoma and acute myeloid leukemia: recent advances

**DOI:** 10.3389/fimmu.2023.1170968

**Published:** 2023-05-05

**Authors:** Jile Liu, Yi Zhang, Ruiting Guo, Yifan Zhao, Rui Sun, Shujing Guo, Wenyi Lu, Mingfeng Zhao

**Affiliations:** ^1^ Department of Hematology, First Center Clinic College of Tianjin Medical University, Tianjin, China; ^2^ Department of Hematology, School of Medicine, Nankai University, Tianjin, China; ^3^ Department of Hematology, Tianjin First Central Hospital, Tianjin, China

**Keywords:** CD7 CAR T-cell therapy, base editing, protein blocker, American society of hematology, natural selection CD7 CAR T-cells

## Abstract

The high expression of CD7 targets in T-cell acute lymphoblastic leukemia (T-ALL) and T-lymphoma has attracted considerable attention from researchers. However, because CD7 chimeric antigen receptor (CAR) T-cells undergo fratricide, CD7 CAR T-cells develop an exhaustion phenotype that impairs the effect of CAR T-cells. There have been significant breakthroughs in CD7-targeted CAR T-cell therapy in the past few years. The advent of gene editing, protein blockers, and other approaches has effectively overcome the adverse effects of conventional methods of CD7 CAR T-cells. This review, in conjunction with recent advances in the 64^th^ annual meeting of the American Society of Hematology (ASH), provides a summary of the meaningful achievements in CD7 CAR T-cell generations and clinical trials over the last few years.

## 1 Introduction

Chimeric antigen receptor (CAR) T-cell therapy is a novel cell-based immunotherapy that has attracted considerable attention from researchers and healthcare professionals due to its outstanding therapeutic efficacy (1). Unlike major histocompatibility complex (MHC)-dependent T-cell receptors (TCRs), CAR can recognize antigens from any MHC background, allowing CAR T cells to target tumor cells that achieve immune evasion through down regulation of MHC expression or impaired proteasome antigen processing (2, 3). CAR T-cells are classified into five generations based on co-stimulatory structural domains, cytokine expression, and transcription factors (4–6). This therapy is typically completed in three steps: physicians first obtain sufficient healthy T cells from the patient or a donor;then, they engineer T-cells ex-vivo using techniques such as lentiviral or electroporation to introduce CARs into the T cells; finally, these modified cells are infused into the patient’s body, where they can efficiently and effectively kill tumor cells by targeting specific antigens (5, 7, 8). CAR T-cell therapy has revolutionized the treatment of hematologic malignancies, particularly in patients with CD19-positive B-cell malignancies, where CD19 CAR T-cell therapy has shown excellent efficacy (9–11). Based on this success, researchers are now seeking to identify suitable targets in other hematological malignancies, such as acute myeloid leukemia, multiple myeloma, and even solid tumor like lung cancer, in order to extend the success of the CD19 CAR T-cell therapy (4, 12–15).In this context, although with the success of anti-CD19 chimeric antigen receptor (CAR) T-cells have successfully treated patients with relapsed/refractory (R/R) B-cell leukemia/lymphoma (9–11). However, the use of CAR T-cells in the treatment of T-cell malignancies is challenging because many targets are co-expressed between normal and malignant cells (16).

**Figure 1:**
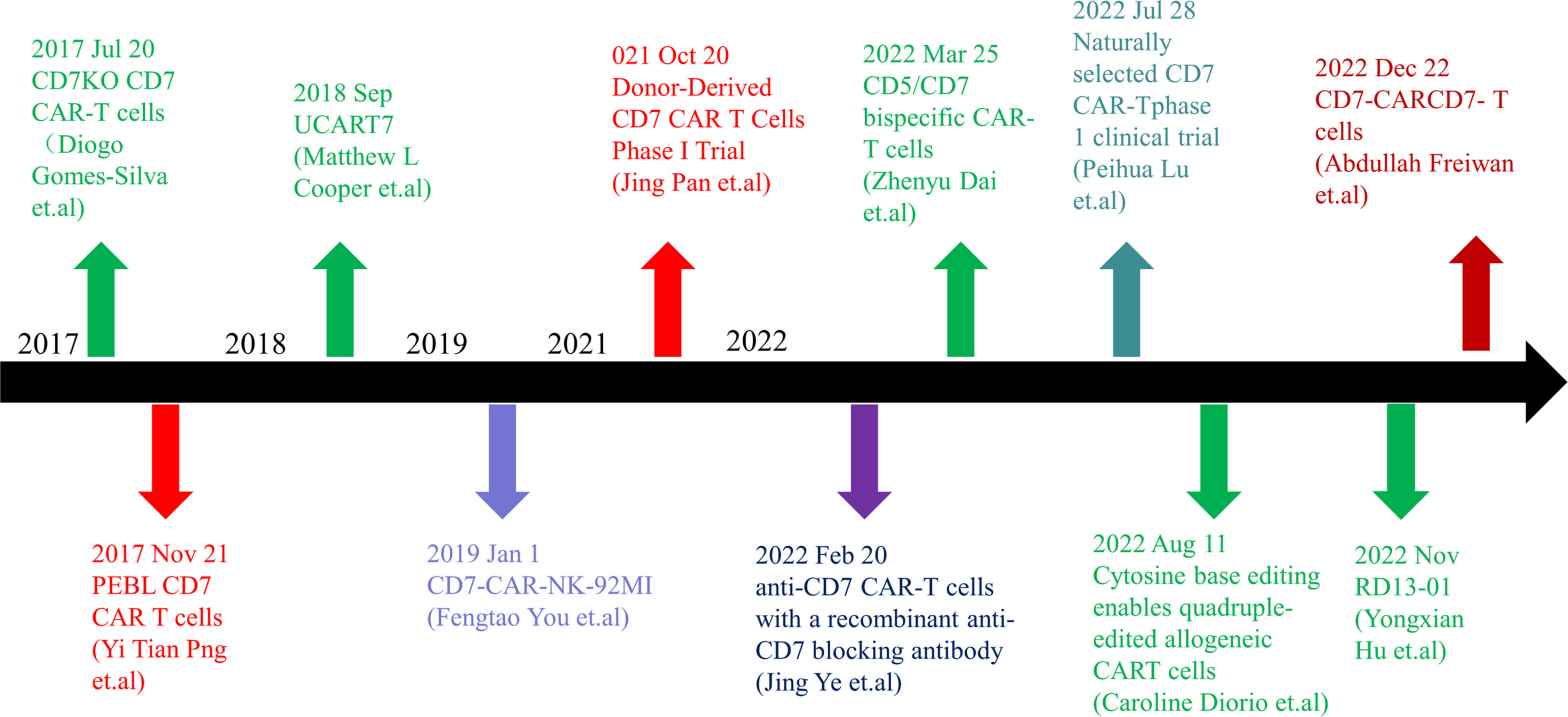
Timeline of CD7 target research. In 2017, anti-CD7 CAR-T cells that can get rid of fratricidal fate were successfully prepared by using gene editing technology and CD7 Protein blocker, which means that the application of CAR-T cells targeted at CD7 targets has become possible in clinical practice and has landmark significance in the research of CD7 targets. In the following five years, there were preparation methods such as allogenic anti-CD7 CAR-T cells, natural selection of anti-CD7 CAR-T cells, preparation of anti-CD7 CAR-T cells by recombinant antibody, and phase 1 and phase 2 clinical trials. With the extension of the time line, many breakthroughs have been made in CD7 target research. CD7 target research is still ongoing, and more successful patients will benefit in the future.

The surface receptor CD7 is a cell membrane glycoprotein with a molecular weight of 40 kDa (17).As a member of the immunoglobulin supergene family, it is an important target for hematological immunotherapy (18, 19). CD7 is considered as a key factor in the treatment of T-cell acute lymphoblastic leukemia (T-ALL) and T-lymphoma due to its widespread distribution on tumors. Meanwhile, the expression of CD7 is also observed in 30% of acute myeloid leukemia (AML) (20, 21),and plays a critical role in the treatment. In fact, CD7 expression is associated with more progressive disease and worse prognosis in these 30% of AML cases. Increased drug resistance may result from positive CD7 expression (22–24). In view of the widespread expression of CD7 in these acute diseases, the importance of CD7 has been the focus of scientific attention from a very early stage. Frankel AE et al.(1997)prepared anti-CD7-dgA (consisting of a deglycosylated ricin A chain coupled with mouse monoclonal anti-human CD7 antibody) for treating of T-lymphocyte malignant hematologic tumors and evaluated its efficacy (25, 26). However, the anti-tumor activity of anti-CD7-dgA is limited. CD7-targeted drugs have not achieved significant results in the treatment of patients with T-cell lymphoma (26). CD7 surface antigens can also be detected on normal T lymphocytes and NK cells, as well as progenitors of thymocytes, lymphocytes, and myeloid cells (19, 27, 28). Therefore, the expression of uninhibited CD7 in CD7 CAR T-cells would trigger the above-mentioned fratricidal phenomenon. Meanwhile, infusion of CD7 CAR T-cells into patients could inadvertently deplete T and NK cells, reducing the patient’s immune competence (16). Fratricide of CD7 CAR T-cells still affects their own proliferative function and cytotoxic effects *in vivo* in the context of traditional methods (16, 21). Therefore, it is the main direction for researchers to improve CD7 CAR-T by not expressing CD7 on the surface of CD7 CAR-T cells to avoid fratricide (**Figure 1**). Concerning this orientation, recent attempts have been made in the fields of gene editing, protein blockers, natural selection, and also some other aspects to improve the property of CD7 CAR-T in promoting the treatment of T-lymphocyte tumors. All these aspects will be critically analyzed from the viewpoints of preclinical experiment and clinical trial, followed by a comprehensive discussion.

## 2 Gene editing CD7 CAR T-cells

In order to remove the mechanism of fratricide (serving as a significant side effect), the gene editing methods mainly aim to carry out the knockout of the gene modulating CD7 expression, while preserving the normal development, proliferation, and the function of producing normal lymphoid organs and immune responses, which has been positively demonstrated by animal experiments (19, 29–31).

### 2.1 CRISPR/CAS9 gene-edited universal CD7 CAR T-cells

Clustered regulatory interspaced short palindromic repeats/CRISPR associated nuclease 9(CRISPR/Cas9) system is a powerful genome editing tool originally adapted from the genetic defense mechanism of info-prokaryotes to self-protect themselves from foreign genetic material (32). It consists of two components: the Cas9 endonuclease, which cleaves the DNA, and a guide RNA, which directs Cas9 to specific DNA sequences. By designing guide RNAs that target specific DNA sequences, researchers can use Cas9 to introduce double-strand breaks into DNA, resulting in gene knockouts or targeted modifications. As a prerequisite, the system requires the presence of a PAM adjacent to the target DNA sequence to function effectively (33).This system is classified into three types based on the presence of different effector complexes. The type II system, which uses a single Cas protein, is the most commonly used for genome editing due to its simplicity and precision (33, 34).

#### 2.1.1 Preclinical experiments

**Figure 2:**
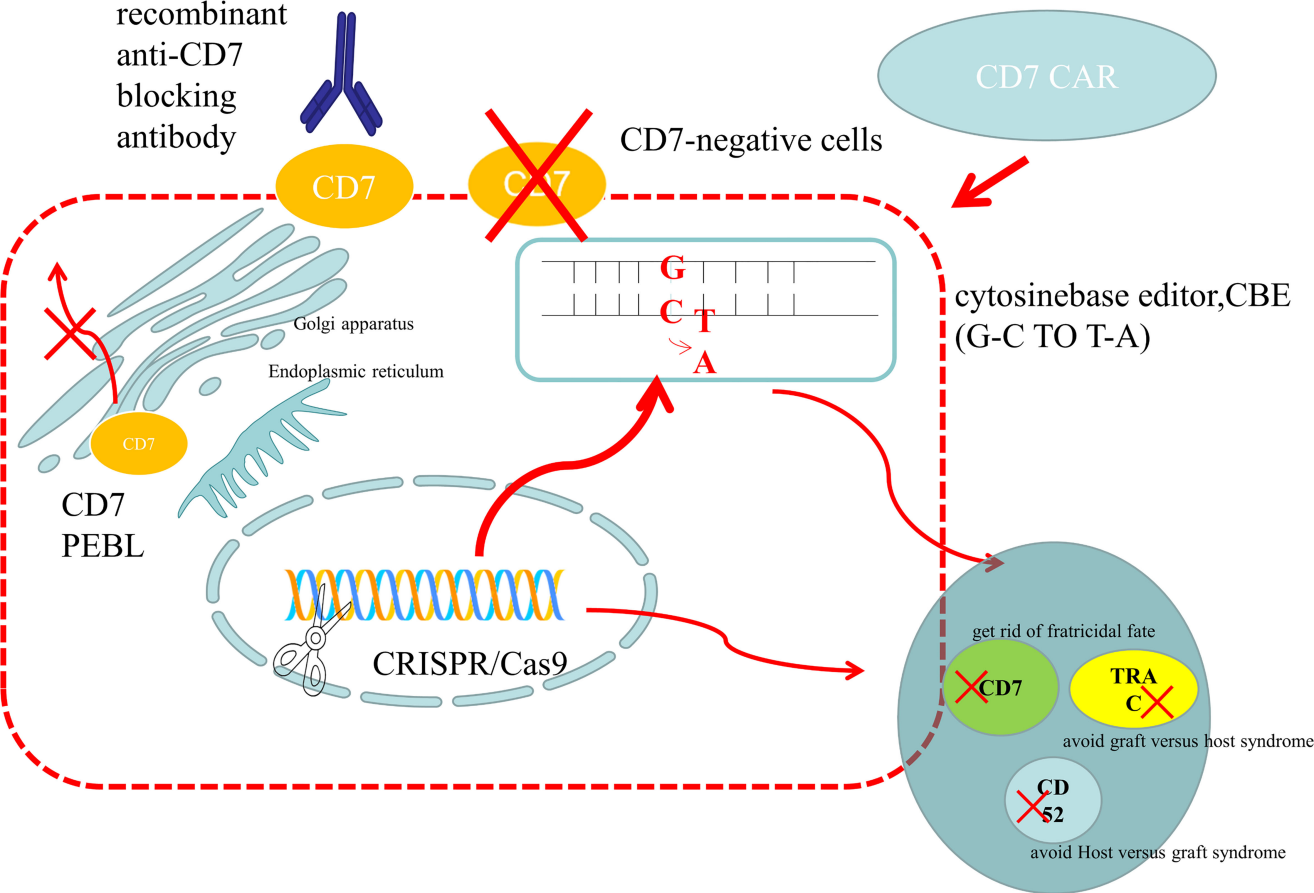
Preparation methods of anti-CD7 CAR-T cells. (1) Use gene editing technology to knock out the CD7 gene to avoid being killed because of the same expression of CD7 protein on the surface of CAR-T cells. For donor-derived T cells, it is also necessary to knock out the TRAC gene to avoid TCR on the surface of T cells and attack the recipient cells, leading to graft versus host syndrome. The purpose of CD52 gene knockout is to use CD52 monoclonal antibody to suppress the host immune system, while retaining the CAR-T cells that are CD52 negative, so that the CAR-T cells can be protected from the attack of host immune cells and ensure their proliferation. At present, the main gene editing used is CRISPR/CAS9, which cuts double-stranded DNA like scissors to achieve the goal of gene knockout. There is also a new gene editing technology – base editor, which improves the security by replacing bases without double strand breaks. At present, both gene editing techniques have been applied to the preparation of anti-CD7 CAR-T cells and have entered the clinical trial stage. (2) The CD7 protein is anchored on the endoplasmic reticulum and Golgi apparatus by CD7 protein inhibitor, which prevents it from being expressed on the cells surface and avoids the emergence of cannibalism. (3) Natural CD7 negative T cells were selected to prepare anti-CD7 CAR-T cells. And natural selection of viable anti-CD7 CAR-T cells through placement were selected. (4) The free anti-CD7 antibody containing the same binding domains as CAR was selected to block the CD7 antigen on the surface of T cells to avoid fratricidal in the preparation of anti-CD7 CAR-T cells.

In this method, CRISPR/CAS9 is used to remove the gene that modulates CD7 expression to prevent CD7 CAR T-cells from fratricide. [cell line: CCRF-CEM; animal model: male and female NGS mice] (16, 35). Since this knockout step is able to preserve the natural functions of CAR T-cells, previous studies have shown a significant viability of CD7 CAR T-cells in the relevant therapies. With this higher viability (compared with traditional methods), the engineered CD7 CAR (CD7^KO^ CD7 CAR) T-cells are more capable of proliferating (16, 36). At the same time, it also performs well in a more potent and specific anti-tumor activity against malignant T cells. Consistent with the revealed mechanism, a protective effect has been observed in an *in vivo* experiment using T-ALL mice xenograft model (4). In this sense, CD7 CAR T-cells have generally shown promise in the treatment of malignant T lymphocytic cancers (**Figure 2**).

The first preclinical data on the effective treatment of T-cell malignancies with universal CAR-T therapy was reported by Matthew L. Cooper et al. (35) Universal CAR T-cells (UCAR-T cells) are typically made from T-cells donated by a healthy donor. The generation of UCAR T cells has the potential to overcome many of the disadvantages associated with the second-generation autologous CAR T cells currently in use: 1. The difficulty of obtaining sufficient healthy T cells from patients for the preparation of CAR T cells (37); Patients with malignancies often undergo multiple rounds of chemotherapy, radiation therapy, and immunotherapy, which can significantly reduce the viability and number of T cells compared to healthy individuals; 2. The lengthy process of CAR-T preparation. Extracting T-cells from the patient’s body and reintroducing CAR-T cells into the body usually takes more than ten days. Due to disease progression, some patients may miss the optimal time for CAR T-cell therapy. Donor T cells can shorten the waiting time and are particularly beneficial for patients with rapidly progressing disease; 3. With autologous collection, there is a risk that T cells collected from patients with T-cell lymphoma may be contaminated with cancer cells (37, 38); T cells collected from patients with malignant tumors in the lymphatic system may not be healthy T cells, but rather malignant tumor cells (39); 4. The cost of preparing autologous CAR-T cells is also unaffordable for patients to shoulder (40). UCAR-T cells has achieved the goal of mass production of CAR T-cells and reducing the difference of autologous preparation. However, there are some problems with the universal CAR T-cells: 1. The host immune system rejects allogeneic T cells; 2. Graft versus host disease (GvHD) of donor T cells against the host (40). To address these issues, researchers used gene editing to knock out the T cell receptor (TCR) gene, the human leukocyte antigen (HLA) class II gene, and CD52 (41). Using gene editing technology to knock out CD52, CAR T-cells can tolerate CD52 antibodies (42). Considering that CD52 is ubiquitously expressed on the surface of lymphocytes, monocytes, and various hematopoietic cells, the property created by the gene editing process could be exploited by clearing the host T-cells. Prior to the administration to allogeneic CAR T-cells, CD52-targeting agents such as alemtuzumab could be administered to aid to the clearance of host T-cells, thereby preventing host versus-graft reactions (HVGR) that may arise result from host T cell-mediated attack on the allogeneic CAR T-cells. Meanwhile, CAR T-cells could also maintain their viability in this environment with these targeting agents (43). 2022 American Society of Hematology Annual (ASH) meeting published that the universal CD7 CART edited by CRISPR/Cas9 could effectively proliferate and specifically kill T-ALL tumor cells *in vitro* (44). It can also significantly reduce the tumor burden and extend mice survival time (cell lines: Jurkat; animal model: NSG mice). Meanwhile, knocking out CD7 related genes also leads to an increase in the CD4 memory cell group without affecting the function of CAR T-cells. In Dai et al. (2022) (45), preclinical developmental data on dual target dual-targeted CAR T-cells were reported. They have combined the two antigens to achieve a higher treatment efficacy and to expand the applicability of this therapy to a broader patient population. After using CRISPR/Cas9 to knock out the genes that modulate CD5 and CD7 expression, the researchers compared the expression status and tumor-killing efficiency of tandem CARs and dual CARs (cell lines: Jurkat, CCRF-CEM, MOLT-4, SUP-T1, Raji; animal model: 6-week-old female NSG mice). Tandem CARs and Dual CARs are two different designs of construction that combine two targets. Each CAR of Dual CAR-T cells has a complete signaling domain that activates the anti-tumor effect in the presence of any homologous antigen. Tandem CAR is a form of CAR in which two distinct antigen-binding domains are co-expressed in one tandem (46). *In vitro* and *in vivo* experiments have been concluded that tandem CAR-T cells have higher transduction efficiencies and cytotoxic effects. Tandem CAR-T cells are even more advantageous than dual CAR T-cells in preventing relapse due to antigen escape (45). Dai et al. (2022) shed new lights on the application of CD7 CAR T-cells, and broaden the scope of the therapy by also targeting the malignant T-cells with CD5 expression, reducing the likelihood of CD7-negative relapse.

#### 2.1.2 Clinical trials

The researchers used the CRISPR/Cas9 system to disrupt the genes that express CD7 and TCRα in T cells and named the edited anti-CD7 CAR T-cell product GC027, an “off-the-shelf” allogeneic CAR T-cells. Shiqi Li et al. (47) reported two cases of T-ALL patients treated with GC027 to evaluate the efficacy and safety of GC027 at *Clinical Cancer Research* in November 2020. This study found patients with both grade 3 cytokine release syndrome (CRS) (**Table 1**) and neurotoxicity, but without GvHD. As for CRS, they repeatedly applied Ruxolitinib, and found that the repeated use of Ruxolitinib did not affect the efficacy of CAR T-cells’ in treatment and their proliferation. With this observation, it was concluded that Ruxolitinib could play a critical role in the prevention and treatment CRS following CAR T-cell therapy (47). However, this study is statistically underpowered (n=2). In this sense, further clinical trials are highly in need to verify their conclusion.

**Table 1:** CRS grading scale.

Grade	Penn grading scale
Grade1 CRS	The patient has fever ≥ 38.0° C and some nonspecific signs, but no hypotension or hypoxia
Grade2 CRS	The patient has a fever ≥ 38.0° C and low blood pressure, but there is no need for vasopressor drugs. Patients with or without hypoxia only need low flow nasal catheters (≤ 6 L/min) for oxygen administration, or blow by oxygen administration.
Grade3 CRS	The patient has a fever ≥ 38.0° C with hypotension, and has no effect on fluid supplementation while requiring high flow oxygen administration (≥ 6 L/min).
Grade4 CRS	The patient has a fever ≥ 38.0° C with hypotension requiring multiple pressor medications and/or with no other causes of hypoxia requiring positive pressure ventilation disposal.

He Huang et al. eliminatedCD7/TRAC/RFX5-related genes using CRISPR/Cas9 technology (48). The researchers utilized a method to prevent host NK cells from attacking CAR T-cells by attaching NK cell inhibitory receptors to the intracellular domain of the T cell costimulatory protein CD28. To compensate for the lack of CD7, they used gene-editing technology to add CD132 to the CAR T-cells to increase the production of IL-2 (49), which enhanced the proliferation and anti-tumor efficacy of the CAR T-cells (cell line: Jurkat; animal model: NSG mice). The Phase I clinical trial used a single-arm, open-label, dose-escalation (Level 1: 1×10^7^cells/kg; Level 2: 2×10^7^cells/kg; Level 3: 3×10^7^cells/kg) design to evaluate the safety and tolerability of CD7-targeting CAR-T cells (RD13-01), and to observe its anti-tumor activity and pharmacokinetic properties. The experimental results show that RD13-01 has high safety and anti-tumor activity with no dose-limiting toxicity (DLT), GvHD and ≥ 3 CRS events. The overall remission rate (ORR) was 82% and 6 leukemia patients achieved minimal residual disease (MRD) (-), with some patients achieving the level of complete response (CR)/complete response with incomplete hematologic recovery (CRi) (48). At this ASH meeting, the Phase I clinical trial of RD13-01 infusion in 10 enrolled patients was reported (NCT04620655) (50). On the 28th day of RD13-01 infusion (low does: 0.5-1×10^7^cells/kg; medium does: 2×10^7^cells/kg; high does:4×10^7^cells/kg), 8 patients achieved complete bone marrow (BM)/peripheral blood (PB), 7 of whom were MRD negative. Only one patient experienced grade 3 CRS, and only one patient experienced grade 3 neurotoxicity. However, some patients died during follow-up (including from disease progression and bacterial infection). Based data from the Phase I trial, the investigators concluded that RD13-01 product is safe and has a dose-dependent effect in the treatment of patients with T-ALL/LBL following high-dose pretreatment. However, long-term follow-up is required and more patients are needed to further evaluate the safety and efficacy of CD7 UCAR-T cells.

### 2.2 Base editor

Although CRISPR/Cas9 is widely used, the mechanism of DNA double-strand breaks (DSBs) often leads to unpredictable adverse outcomes. For example, complex genome rearrangements and high-frequency translocations that are caused by the simultaneous induction of multiple DSBs (51–53). Considering that the preparation of UCAR-T requires several editions before the final application in clinical practice, DSB-based gene editing technology may have a negative impact on the efficacy and safety of universal CAR-T therapy (51–54). In recent years, the base editing technology has been gradually developed and matured. Compared with CRISPR/Cas9, the base editor does not need to break the double-stranded DNA, so it has higher safety and accuracy (55, 56). Cytosine base editors (CBEs) are made by fusing cytidine deaminase into Cas9 endonuclease (nicase) or TALEN sequence with uracil glycosylase inhibitory domain (UGI) to convert C • G base pairs to T • A base pairs at specific locations in the genome (57). Adenine base editors (ABEs) are another common type of base editor that facilitates the conversion of A•T base pairs into G•C base pairs (58). Another variant of base editors, the TadA-derived cytosine base editors (TadCBEs), have also been developed and shown to be effective for precise gene editing (59). To date, it has been demonstrated that TadCBEs can be used for efficient multi-site cytosine base editing of treatment-related targets in primary human T-cells, and cytosine base editing of treatment-related sites in primary hematopoietic blood stem cells and progenitor cells (HSPCs).

#### 2.2.1 Preclinical experiments

Diori used CBE to develop a clinically acceptable quadruple-base-edited allogeneic CAR T-cell therapy targeting CD7 (7CAR8) for the treatment of T-ALL (60). 7CAR8 has blocked the expression of CD52, CD7, PD1 and TCRα protein. Their preclinical experimental results indicate that 7CAR8 avoids the challenge of collecting T cells from T-ALL patients because T cells are obtained from healthy donors. And it reduces the possibility of fratricide in CAR T-cells, GVHD, and receptor rejection of allogeneic CAR T-cells. Meanwhile, this study also proves that the CAR T-cells prepared in this way can effectively and safely eliminate tumor cells *in vitro* and *in vivo* (cell line: CCRF-CEM; animal model: NGS mice) (60).

In this ASH meeting, the excellent property of base editing has been demonstrated in the removal of TCR, CD52, the common AML/T lineage antigen CD7 (61). It produced the BE-CAR33 T-cells and BE-CAR7 T-cells originally obtained from donor sources, targeting the AML with CD7 and CD33 positive. *In vivo* experiments have shown that, in NGS mice, compared with the control group, which goes with the CAR T-cells targeting CD19 and CD7, AML cells (Molm14 and Kasumi) in the group with cells targeting only CD33 and the group with a combination of targeting CD33 and CD7 significantly decreased. The survival rates of mice treated with BE-CAR33 T-cells and BE-CAR33/BE-CAR7 T-cells were significantly prolonged. CAR T-cells showed strong persistence. This experiment demonstrated the reliability and efficacy of the combination of BE-CAR33 T-cells and BE-CAR7 T-cells for the treatment of AML.

#### 2.2.2 Clinical trials

In 2022, the CD7 CAR-T basic editing clinical trial (ISRCTN15323014) was initiated (62). The CAR T-cells were administered prior to allogeneic stem cell transplantation. Eligible patients were pretreated with fludarabine, cyclophosphamide and alemtuzumab to promote lymphodepletion, and then infused with 0.2-2.0×10^6^ BE-CAR7 T-cells. Patients in remission on day 28 underwent allogeneic stem cell transplantation to deplete BE-CAR7 T-cells and promote immune reconstitution. The Phase I study is designed to treat 10 children. One child has been enrolled currently. After receiving BE-CAR7 T cells, the patient observed grade 2 CRS and grade 1 ICANS without GvHD. After 28 days, the child showed morphologic remission without count recovery and received low-intensity allogeneic stem cell transplantation.

## 3 Protein blocker

Protein Blocker (PEBL) consists of a single chain variable fragment and an intracellular retention domain that anchors the cognate antigen in the endoplasmic reticulum and Golgi apparatus before degradation. CD7 PEBL is a technique that does not require the downregulation of endogenous CD7 by gene editing (63). This method anchors the CD7 protein in the endoplasmic reticulum and/or Golgi apparatus and prevents it from being expressed on the surface (63). Studies have shown that the retention of CD7 on T cells does not affect their function and proliferation. Therefore, PEBL is also an effective method to produce CAR T-cells without CD7 protein on the cell surface (21, 64).

### 3.1 Preclinical experiments

Since the preparation of autologous CAR T cells and the application of this therapy could depend on the amount of leukemia cells and/or the number of T cells (taking into account the potential contamination and the lower proportion of healthy cells driven by the malignancy), two “universal” allogeneic CAR-T cells prepared by PEBL and lentivirus transduction technology were presented in this 2022ASH. The two teams both used PEBL technology, but to develop CD3 CAR-T and CD7 CAR-T, respectively.

Because peripheral T-cell lymphoma (PTCL) arises from mature T cells, both T lymphocytes and most tumor subtypes both maintain a high persistent CD3 expression. To overcome the drawbacks of this feature, Hongliang Qian’s team used PEBL to downregulate surface CD3 to avoid the fratricide of CD3 CAR T-cells (65). They developed CD3 CAR T-cells for the treatment of PTCL, which showed excellent property in *in vitro* (CCRF-CEM T-ALL cell line) and *in vivo* experiments in mice.

In Xing Fah Alex Wong’s team, the feasibility of the simultaneously using of two PEBLs for intracellular protein retention was well validated, indicating that the key functions of CAR T-cells were not affected. They developed the anti-CD7 CAR T-cells depleted of the CD7-CD3- (PCART7) expression for the treatment of R/R T-ALL or T-cell lymphoblastic lymphoma (66). To generate TCR/CD3-deficient PCART7 cells from healthy donor T-cells, a double transduction method is used. The method involves the use of two lentiviral vectors, the first vector being bicistronic and carrying CD7 PEBL and anti-CD7 CAR, while the second vector carries CD3 PEBL. In this study, more than 90% of the cells have displayed the phenotype of CAR+CD7^-^CD3^-^. After screening and purification, this number could be increased to 99%. This type of preparation for CAR T-cells shows the same effect as the genetically modified knockout of TRAC and CD7, while avoiding the risk of gene translocation and rearrangement. The potent cytotoxicity of CAR-T against CD7^+^ leukemia cells was confirmed in both short-term and long-term *in vitro* assays. In the xenograft model using the CCRF-CEM T-ALL cell line, PCART7 effectively inhibited tumor growth and extended mice survival time.

### 3.2 Clinical trials

In one clinical trial, CD7 CAR T-cells derived from autologous nanoantibodies were used to treat R/R T-ALL/LBL (67). A CD7 blocking strategy was developed using a tandem CD7 nanoantibody VHH6 coupled to the endoplasmic reticulum/Golgi retention motif peptide to immobilize CD7 molecules in cells. Preclinical studies have shown that CAR T-cells are not fratricidal and exert potent cytolytic activity, significantly attenuating leukemia progression and extending the survival time of mice in NPG mice injected with Luc+ GFP+CCRF-CEM cells. Eight patients were subsequently enrolled in a clinical trial (NCT04004637). Clinical trial results showed that seven patients achieved CR after 3 months of CAR T-cell infusion, with most patients experiencing only grade 1 or 2 CRS and no T-cell aplasia or neurotoxicity (67). This CAR T-cell therapy merits further study in highly invasive CD7-positive malignancies.

In 2021, Pan Jing’s team published the results of the Phase I clinical trial of CD7 CAR T-cells from PEBL-treated donors in the *Journal of Clinical Oncology (* 21 *).* 20 patients were enrolled in the Phase I clinical trial. Patients received high-dose pretreatment chemotherapy prior to the CAR T-cell infusion [5 × 105 or 1 × 106 ( ± 30%) cells/kg] with no subsequent DLTs. Grade 3 or higher CRS only occurred in only 10% of patients, and neurotoxicity is mild and self-limiting. Early GvHD occurred in 60% of the subjects, with a mild and controllable manifestation. The therapeutic effect in up to 90% of patients demonstrating CR is accounted for by CD7 CAR T-cells. Despite their allogeneic nature, CAR T-cells proliferate effectively in all patients and can be maintained in the absence of SCT, without evidence of rejection (21). The first phase of the trial was only conducted with only a single target dose of 1×10^6^/kg proven to be safe and effective. However, the sample size of this study was not statistically powerful, and the participants were also followed for a relatively short period of time. Therefore, the long-term efficacy and toxicity of this therapy could not be determined in this study.

At the 2022 ASH, the PAN team presented an interim report from the Phase II clinical trial of donor-derived CD7 CAR T-cells for the treatment of R/R T-cell acute lymphoblastic leukemia/lymphoma (NCT04689659) (68). The interim analysis was performed when the first 20 patients who received CD7 CAR T-cells in the Phase I clinical trial completed or discontinued the infusion at the point of 3-month point after the start of the infusion. At the same time, additional patients with mediastinal malignancies were enrolled in the Phase II trial in addition to the original sample. The best overall response rate (BOR) at 3 months was 90%, with only two (10%) patients developing grade 3 or higher CRS, and eight (40%) patients developing grade 1-2 GvHD. However, it should be noted that these side effects were reversible, while the CD7 negative relapse was considered the most important issue affecting survival in this study.

In this sense, they also proposed an alternative in response to these adverse effects. Namely, the infusion of CD5 CAR T-cells, about which they also reported the Phase 1 clinical trial in treating five patients with negative recurrence after CD7 CAR T-cell therapy on the 2022 American Society of Clinical Oncology (ASCO), displaying satisfactory results (69). This trial demonstrated the potential of CD5 CAR T-cells as a subsequent therapy for CD7 negative relapse Jia Feng et al. (70) generated CD5 CAR T-cells that are specifically capable of secreting interleukin 15(IL-15). Meanwhile, this type of CD5-IL15/IL15 sushi CAR may have a beneficial influence on treating T-cell malignancies that have metastasized to the central nervous system (71).

## 4 CD7 CAR^CD7−^ T cells and natural selection CD7 CAR T-cells

The studies presented in the previous sessions mainly consider challenges for CD7-targeted immunotherapies posed by cell fratricide and eradication. However, whether using genome editing to modify the CAR T-cell gene or PEBL to restrict the expression of CD7 protein on the CAR T cell membrane, although all are reported to have avoided potential adverse effects, the possibility of compromising the normal physiological function of CAR T-cells being affected still exists as the “intact” declarations are all preliminary and based on statistically weak probabilistic evidence. Therefore, the present section will mainly focus on the studies innovating in the preparation of CD7 CAR T cells in a more natural way to shed light on the absolute neutral effects on the cell itself, emphasizing on the mechanism of the resistance to the fratricide while limiting the use of cell modification, covering CD7 CAR^CD7−^ T cells and natural selection CD7 CAR T-cells.

### 4.1 Preclinical experiments

In response to the above-mentioned issues, natural or manually selected CD7^-^ T-cells have come into the scientific focus as be a promising cell source for the production of CD7^-^CAR T-cells (72). CD7 is involved in transcriptional regulation and the lack of CD7 messenger RNA results in a stable distribution of CD7^-^T-cells. Naturally occurring CD7^-^T-cells may be a promising cell source for the generation of CD7^-^CAR T-cells. These CD7-T cells primarily have a CD4+ memory phenotype and typically display a Th0/Th2 phenotype after transplantation or in other immunodeficient environments. Researchers have found that CD7-negative T cells exist in the peripheral blood of healthy donors (0.72% to 19.5%) as well as patients with T-cell acute lymphoblastic leukemia (T-ALL) and B-cell acute lymphoblastic leukemia (B-ALL) (3% to 12.5%).

Some researchers screened out naturally occurring CD7^-^ T-cells by 2-step magnetic bead separation to generate CD7 CAR^CD7-^ (72). Compared with the CD7 CAR T-cells that did not go through this process, no fratricide was reported in the production and proliferation process of the CD7 CAR^CD7-^ T-cells that went through the selection. At the same time, the studies found that CD7 CAR^CD7-^ T-cells have rich CD4^+^effect memory phenotype, maintaining their ability in cytotoxic activity and cytokine secretion, and a stably lower expression of checkpoint inhibitory receptor. In addition, CD7 CAR^CD7-^T-cells demonstrated *in vivo* persistence and protection of NGS mice receiving 1×10^4^ CCRF (T-ALL) or 3×10^6^ BV173 (B-ALL) cells from tumor relapse. The CD7 CAR^CD7-^ T-cells screened showed superior anti-tumor function and durability compared to conventional CAR T-cells, and a distinct transcriptional activation spectrum. However, access to sufficient healthy CD7-T cells in the peripheral blood is still a challenge, which also promotes difficulties in the subsequent selection, and overall proliferation and overall production of T-cells to meet the demands of this type of therapy (72). Therefore, the feasibility of studies in this pathway still remains to be tested.

Unlike the CD7 CAR^CD7-^ T-cells, Lu Peihua’s team employed a natural screening method to prepare the CD7 CAR T-cells. No other operations were performed during the preparation of the CD7 CAR-T. The main move of this method is to place the generated CD7 CAR-T cells in a natural state without restricting fratricide to acquire the final surviving cells (48) Screened out by this targeted natural selection, these CD7 CAR T-cells demonstrated high therapeutic efficacy in both T-lymphocyte malignancies and AML, contributed by the high level of CAR and CD7 negative expression *in vitro*. This manifestation stands out as an excellent prominence in the context that normally 20-35% of AML patients have high CD7 expression, leading to a higher likelihood of poor prognosis (73–75). Although a proliferation fatigue and higher cell death have been reported after the fratricide screening, the amount of target cells is still sufficient for the dose required for transfusion back to patients to complete the therapy. Notably, although the CD7 receptor T-cell defect was caused, the CD7^-^ T-cell subset simultaneously shouldered the main function of the first one, alleviating the treatment-related T-cell immune deficiency. After subsequent allotransplantation, the number of NK-cells and T lymphocytes can quickly return normal. Finally, the CD7 CAR T-cell depletion marker analysis of natural selection was performed, which showed that the expression of PD-1 and TIM-3 were reported with a statistically significant increase. This indicates that long-term placement may cause CAR-T cells to enter a depleted state, resulting in decreased anti-tumor activity and proliferation of CAR T cells after their re-export into the body. During the manufacturing process of NS7CAR-T cells, there is a risk that the final number of CAR T cells may not meet the standard for transfusion due to excessive self-killing of CAR T cells. These factors may limit the widespread use of NS7CAR-T cells.

### 4.2 Clinical trials

In 2022, Lu Peihua’s team published the results of the clinical trial of the CD7 CART (NS7CAR) on the natural selection in *Blood (* 38 *).* The results of the Phase I clinical trial are encouraging. Of the 20 patients selected in the Phase 1 trial (low dose: 0.5×10^6^/kg; medium dose:1 to 1.5×10^6^/kg; or high dose:2×10^6^/kg), 19 patients achieved MRD-negative CR in BM at day 28 and only 1 patient had grade 3 CRS. At this ASH, Lu posted the long-term observation results of his Phase I/II clinical trial. Natural selection targeting CD7 CAR-T (NS7CAR-T) cells eliminates the need for gene editing, protein blockers and other technologies, and greatly reduces the cost of preparation. NS7CAR therapy includes 4-1BB and CD3ζ second-generation murine CAR-T with a costimulatory domain. A total of 53 patients were enrolled in the study. At day 28, 95.8% (46/48) of patients achieved MRD (-) CR) in BM/PB. In 53 patients, the 18-month overall survival (OS) and event-free survival (EFS) rates were 75.0% and 53.1%, respectively. 32 patients were bridged with allogeneic HSCT within 3 months, and OS and EFS at 18 months were 75.8% and 71.5%, respectively. Mild CRS occurred in 47/53 (88.7%) patients. Five patients developed Grade III CRS and one patient developed Grade IV CRS. Grade I neurotoxicity was observed in only 2 patients. Their Phase I/Phase II study showed that NS7CAR was safe and effective in R/R T-ALL/LBL patients receiving high-dose pretreatment, including those with extramedullary involvement and a history of allogeneic HSCT (**Tables 2**, **3**).

**Table 2:** Safety and effectiveness of clinical trial of CD7 CAR T-cells.

	Clinical Trial [Reference]	Phase	Costimulatory Domain	Source of CAR T-cells	The Number of Patients	The Doses of Infusion	Response Status	CRS(≤ Grade2)	CRS(≥Grade3)	CRES/ICANS	Other Adverse Effects
CRISPR/CAS9	NCT04538599 [ (48)]	I	Unknown	Healthy donors	12	1×10^7^ cells/kg; 2×10^7^ cells/kg;3×10^7^ cells/kg	On the 28th day after the infusion, 7/11 patients had CR/CRi. (One patient died of septicemia.)	10	0	0	CMV/EBV reactivation; Neutropenia; Septicemia
CRISPR/CAS9	NCT04620655 [ (50)]	I	41BB	Healthy donors (Universal CAR T-cells)	10	0.5-1×10^7^cells/kg; 2×10^7^cells/kg;4×10^7^cells/kg.	On the 28th day after the infusion, 8/10 (80%) patients’ BM/PB achieved CR/CRi, two patients still NR in the low dose group.	9	1	1	None
Base-Editing	ISRCTN15323014 [ (62)]	I	/	Healthy donors (Universal CAR T-cells)	1	0.2-2.0×10^6^ BE-CAR7 T-cells	Bone marrow evaluated at day 28 showed morphological response.	1	0	1	No GvHD
PEBL	NCT04004637 [ (67)]	I	/	Patients themselves	8	One or two infusions, 0.5-5×10^6^/kg	Three months after CAR T-cells infusion, the CR rate was 87.5% (7/8).	7/8	1/8	0	Abdominal infection; Thrombocytopenia; Moderate anemia
PEBL	ChiCTR2000034762 [ (21)]	I	41BB	Healthy donors	20	1×10^6^( ± 30%) cells/kg. The patients allowed to receive low-dose infusion of 5 × 10 (± 30%)/kg if CAR T-cells did not reach the target dose.	Before receiving any other treatment, 19 patients were in response and 18/19 patients achieved CR.	18/20	2/20	3/15	GvHD; Anemia; Thrombocytopenia; Neutropenia; Lymphopenia; Viral-infection
PEBL	NCT04689659 [ (68)]	I	41BB	Healthy donors	20	1×10^6^( ± 20%) cells/kg.	The BOR rate was 90% at 3 months.	/	2/20	3/20	GvHD; Fulminant hepatitis; Pneumonia; Severe infection
Natural selection	NCT04572308 [ (38)]	I	CD28TM-41BB	Healthy donors or patients themselves	20	Low dose: 0.5×10^6^/kg;mid-dose:1~1.5×10^6^/kg; high-does: 2×10^6^/kg.	19 patients obtained MRD (-) CR in BM on the 28th day.	18/20	1/20	2/20	Bacterial infection; CMV reactivation; Anemia; Neutropenia; Thrombocytopenia; Lymphopenia
Natural selection	NCT04572308&NCT04916860 [ (76)]	I/II	41BB	Healthy donors (2) or patients themselves (51)	53	Low dose: 0.5×10^6^/kg;mid-dose:1~1.5×10^6^/kg; high-does: 2×10^6^/kg.	At day 28, 46/48 of the BM affected patients achieved MRD (-) CR in BM/PB.	47	5	2	None
The fourth generation CAR T-cells	NCT04033302 [ (77)]	I	CD28-41BB-caspase 9 genetic-cassette	patients themselves	1	2×10^6^ cells/kg.	At day 28, the patient achieved CR.	Grade 1 CRS	None	None	Neutropenia

CAR T-cells, chimeric antigen receptor T-cells; CRS, cytokine release syndrome; CRES, CAR-T Cell-related Encephalopathy Syndrome; ICANS, Immune Effector Cell-Associated Neurotoxicity Syndrome; CMV, Cytomegalovirus; EBV, Epstein-Barr virus; GvHD, Graft versus host disease; BM, bone marrow; PB, Peripheral blood; CR, complete response; CRi, complete response with incomplete hematological recovery; PEBL, Protein blockers; BE-CAR7 T-cells, base-editing CD7 CAR T-cells; BOR, Best overall rate; MRD, Minimal residual disease.

**Table 3:** Transplant status and various disease responses in clinical trials.

		T-ALL	T-LBL	AML	Number of bridge transplants	Post-transplant situation
(48)	**Number of people**	7	4	1	4	4CR
	**Day28 evaluation**	5CR	1CR,2PR	CR		
(21)	**Number of people**	20	0	0	7	6CR (One patient died 14 days after SCT from GvHD).
	**Day28 evaluation**	18CR 1PR				
(38)	**Number of people**	14	6	0	10	7CR
	**Day28 evaluation**	13CR/CRi	6CR/CRi			
(77)	**Number of people**	1	0	0	1	CR
	**Day28 evaluation**	CR				

T-ALL, T-cell acute lymphoblastic leukemia; T-LBL, T-cell lymphoblastic lymphoma; AML, Acute myeloid leukemia; CR, complete response; CRi, complete response with incomplete hematological recovery; PR, partial response.

## 5 Preparation of anti-CD7 CAR-T cells using recombinant anti CD7 blocking antibodies

In 2022, a research team proposed and demonstrated the feasibility of a new strategy to generate anti-CD7 CAR T cells using recombinant anti-CD7 blocking antibodies. To avoid the gene toxicity caused by genome editing and the unknown biological function caused by the lack of CD7 expression on the cell membrane, they selected free anti-CD7 antibodies containing the same binding domain as CAR to block the CD7 antigen on the surface of T cells, so as to avoid fratricidal killing during the preparation of anti-CD7 CAR-T cells. They demonstrated that anti CD7 CAR-T cells cultured with antibodies during the preparation phase had higher cell viability and proliferative capacity, and harvested sufficient numbers of expected anti CD7 CAR-T cells. This provides a rapid and safe method for the preparation anti-CD7 CAR T cells, which deserves in-depth research and attention for subsequent clinical trial results (78).

## 6 Comparison of the effects of auto- and allo-CAR T-cells

At the 2022 ASH meeting, the comparison of clinical efficacy, durability and safety of two types of autologous and donor CD7 CARTs was published (NCT04823091) (79). The costimulatory domain of the CAR-T is 4-1BB. The study has just enrolled 10 patients. Five patients were randomly assigned to receive autologous CAR T-cells and the other five to receive allogeneic CAR T-cells. Efficacy and safety comparisons from this clinical trial are shown in **Table 3**. During the follow-up period, 50% of the patients (4/8) showed a relatively high level of CAR-T by qPCR at month 2, of whom 3 received allogeneic CAR-T cells and 1 autologous CAR-T cells. They concluded that the selection of the source of CAR T-cells and the appropriate supportive care are key to efficacy. They concluded that the source of the CAR T-cells and the appropriate supportive care are the keys to good efficacy. The risk of relapse is higher after treatment with autologous CAR T-cells. Therefore, consolidation therapy is necessary. Because donor-derived CAR T cells may increase the likelihood of rejection, infection, it is necessary to maintain long-term detection to achieve better efficacy (**Table 4**). The sample size of this study is only 10 people, and it is still necessary to increase the sample size for further analysis and comparison to ensure the authenticity of the results.

**Table 4:** Comparison of the safety of autogenous and allogenic CAR T-cells in this clinical trial.

	Recurrence rate	Serve CRS	GvHD	Infection	Thrombocytopenia (median time)	Viral infection
autogenous	100%	0%	20%	60%	25days	40%
allogenic	25%	20%	20%	20%	28days	20%

CRS, cytokine release syndrome, Serve CRS≥Grade 3; GvHD, Graft versus host disease; BM, bone marrow.

## 7 CD7 CART cell therapy for MPAL

Mixed phenotype acute leukemia (MPAL), a rare malignancy among acute leukemias, can cause multiple organ failure in patients. MPAL is typically associated with a relatively poor prognosis (80). At the ASH meeting in 2021, the clinical trial of CD7 CAR-T therapy for R/R CD7-positive MPAL patients was published to verify the safety and efficacy of the treatment (80). The investigators selected the second-generation CD7 CAR T-cells with 4-1BB costimulatory domain to treat 4 patients with MPAL and 1 patient with FLT3 mutation. The patients were infused with different doses of CD7 CAR T-cells. Four weeks after infusion, four-fifths of these patients achieved either CR or CRi in the bone marrow, and all achieved MRD-negative CR. This study confirmed the efficacy of CD7 CAR T cell therapy for CD7-positive MPAL, expended the scope of CD7 CAR T-cell therapy and provided new ideas for the treatment of MPAL.

## 8 CD7 CAR-NK

In addition to introducing of anti-CD7 CAR into T cells to generate CD7 CAR T cells, other investigators have also attempted to generate CD7 CAR NK-cells for the treatment of T-lymphocyte malignant blood tumors and have made progress (37). They constructed monovalent CD7-CAR-NK-92MI and bivalent dCD7-CAR-NK-92MI cells using the CD7 nanobody VHH6 sequence and found that they exhibited high efficiency and specific anti-tumor activity on T-cell leukemia cell lines and primary tumor cells. Bivalent dCD7-CAR-NK-92MI monoclonal cells promote granzyme B and interferon γ (IFN-γ) secretion. They demonstrated that CD7-CAR-NK-92MI cells can be used to treat T-ALL. At 2022ASH, researchers presented the experiment of using human invariant natural killer T (iNKT) to generate CD7-CAR iNKT-cells to treat all T-ALL subtypes and 30% of CD7+AML patients. They found earlier. There is a high proportion of CD7-negative cells in iNKT cells from healthy donors. CD7 CAR iNKT-cells prepared by researchers using donor-iNKT-cells are more effective in 70% of CD7^+^CD1d^+^T-ALL patients because they provide dual target specificity and reduce the possibility of relapse (81).

## 9 Discussion

In recent years, CD7 CAR T-cell therapy technology has made significant progress in avoiding or using CAR T-cell fratricide for therapeutic purposes. It has been demonstrated that CAR T-cells can maintain normal physiological functions even when the gene expressing CD7 is deleted, providing a solid basis for the application of genome editing in CD7 CAR T-cells. Using CRISPR/Cas9 genome editing to knock out the CD7 gene has been a feasible method in several preclinical and clinical trials to prepare CD7-targeted CAR T-cells that do not express CD7 on the cell surface. However, the editing of multiple gene loci, which requires different DNA double-strand breaks, may pose a risk of genotoxic side effects. Therefore, a critical evaluation of genotoxic side effects is essential for CD7 CAR-T cells generated by gene-editing techniques using DNA double-strand breaks (DSBs), such as the CRISPR/CAS9 system, to avoid potential risks in subsequent therapies and trials. The advent of base editors has ushered in a new wave of gene editing of CAR T-cells. Base Editors can precisely knock out CD7 and other target genes without fear of adverse effects from DNA double-strand breaks. Base editor-edited universal CD7 CAR T-cells can replace existing CD7 CAR T-cells and hold promise for patients with insufficient healthy T cells or rapid tumor progression. PBEL fixes the CD7 protein in the endoplasmic reticulum and Golgi apparatus from the organelle level, which is safer and more convenient without gene editing. Meanwhile, recent ASH studies have shown that a combination of CD3-PEBL may function similarly to TRAC knockout, making PBEL-generated universal CD7 CAR T-cells safer and reducing the risk of GvHD. New strategies to generate anti-CD7 CAR T-cells using CD7-negative cells, natural selection targeting CD7 CAR T-cells (NS7CAR-T) and recombinant anti-CD7 blocking antibodies have provided us with new ideas. These methods can achieve the desired results under CD7 expression on the surface of CAR T-cells, thus saving costs and avoiding the uncertainty caused by complex operations. Based on the studies reviewed in this article, we can expect more and better preparation methods in the future.

For the study of CD7 CAR T-cell therapy, further research is needed to prepare CAR T-cells from autologous or allogeneic T-cells. The quality of T-cells from patients with malignant T lymphocyte hematologic tumors is poor and easily contaminated with malignant tumor cells. Therefore, the production of CD7 CAR T-cells from healthy donor T-cells may be a better choice. However, donor-derived CD7 CAR T-cells also have many problems, including graft-versus-host syndrome and the risk of genotoxicity caused by gene editing. Whether allogeneic CD7 CAR T-cells can achieve better curative effects remains to be determined. More clinical data are needed to explore the advantages and disadvantages of autologous and allogeneic CAR T-cells and to select the most appropriate source of CD7 CAR T-cells for different situations.

Cell therapy for the CD7 target is still in its infancy and many factors such as efficacy, side effects and relapse need to be evaluated. CD7-negative relapse and infection are currently prominent problems in clinical data. To cope with the negative recurrence of tumor patients after CD7 CAR-T treatment, it may be an excellent solution to find new targetsCD5 CAR T-cells may be a practical choice, and its efficacy in the treatment of T-cell malignant tumors has been verified in past experiments. We hope that CD5 CAR T-cells can become a follow-up treatment for CD7-negative relapse, just like the addition of CD20 CAR T-cells when CD19 CAR T-cells cannot work (82, 83). In addition, anti-CD4 CAR T-cells, anti-T cell receptor beta constant 1 (TRBC1) CAR T-cells and anti-chemokine receptor 9 (CCR9) CAR T-cells have also shown promising effects in preclinical research for the treatment of T-cell malignancies and may become new targets for the clinical treatment of T-cell malignancies in the future (84–86). Dual-targeted CAR T-cells are also a direction in which we can conduct in-depth research to reduce the possibility of tumor escape. A preclinical experimental study of CD5/CD7 CAR T-cells by Dai et al. has given us ideas for further clinical trials. At the same time, we hope to see more trials of double-targeted CAR T-cells in the treatment of T-lymphocyte malignancies. It is also worth exploring how to avoid and manage the risk of infection of CD7 CAR T-cells after treatment. The infusion dose of CD7 CAR T-cells and bridging transplantation, as well as the combination of CD7 CAR T-cells and drugs, may be the focus of future research. We need to continue to explore the process to find a more promising treatment. We have high hopes for CD7 CAR T-cells and hope that they can relieve pain for more patients.

## Author contributions 

JL and YZ are major contributors to writing the manuscript. RG has made substantial contributions to the conception. YZ and JL have drafted the work. RS, YFZ, WL, and SG have substantively revised it. MZ reviewed the draft. All authors contributed to the article and approved the submitted version.
